# 43.1 GENETIC INSIGHTS LEAD TO DISCOVERY OF SELECTIVE ACTIVATORS OF MGLU1 AND MGLU3 METABOTROPIC GLUTAMATE RECEPTORS AS POTENTIAL TREATMENTS FOR SCHIZOPHRENIA

**DOI:** 10.1093/schbul/sby014.179

**Published:** 2018-04-01

**Authors:** P Jeffrey Conn, Samantha Yohn, Branden Stansley, Dan Foster, Hyekyung Plumley, Craig Lindsley

**Affiliations:** 1 Vanderbilt University Medical Center; 2 Vanderbilt University

## Abstract

**Background:**

A large number of clinical and preclinical studies suggest that dysfunction at synapses for the excitatory neurotransmitter glutamate may play a critical role in the pathophysiological changes that underlie each of the major symptom clusters observed in schizophrenia patients. Interestingly, recent genetic studies identified multiple nonsynonymous single nucleotide polymorphisms (SNPs) in the human genes encoding two specific subtypes of metabotropic glutamate (mGlu) receptors that are associated with schizophrenia. These include GRM1 and GRM3, the genes encoding for the mGlu1 and mGlu3 receptor subtypes respectively. Furthermore, postmortem studies suggest that expression of these mGlu receptor subtypes is altered brains of schizophrenia patients compared to controls. Mutations in GRM1 were identified a range of schizophrenia patients, whereas SNPs in the human gene encoding mGlu3 (GRM3) are selectively associated with poor performance on cognitive tests that are dependent on function of the prefrontal cortex (PFC) and hippocampus. These studies raise the possibility that disrupted signaling of mGlu1 and/or mGlu3 could contribute to the symptoms of schizophrenia and that selective modulators of these receptors could provide a novel approach to treatment of this disorder.

**Methods:**

Wild-type and mutant forms of mGlu receptors were expressed in cell lines and used for discovery and optimization of highly selective positive allosteric modulators (PAMs) of mGlu1 and mGlu3. Optimized mGlu1 and mGlu3 PAMs were then used along with mouse genetic studies to evaluate the roles of these receptors in specific basal ganglia and forebrain circuits that have been implicated in schizophrenia. Finally, these compounds were used in animal models to assess potential efficacy in rodent models that are relevant for reducing positive, negative, and cognitive symptoms that are observed in schizophrenia patients.

**Results:**

GRM1 mutations associated with schizophrenia were found to reduce mGlu1 signaling, suggesting that loss of function of this receptor could contribute to symptoms associated with schizophrenia. Furthermore, we found that highly selective mGlu1 PAMs reverse deficits in mGlu1 signaling observed in these mutant receptors, induced a profound reduction in dopamine release in striatal areas implicated in schizophrenia, and have robust antipsychotic-like effects that are mediated by localized inhibition of dopamine release in striatum. In contrast to existing antipsychotic medications, selective mGlu1 PAMs also improve motivation and reduce anhedonia in animal models. Interestingly, selective mGlu3 PAMs have multiple effects in the prefrontal cortex and hippocampus that would be expected to improve cognitive function. Consistent with this, highly selective mGlu3 PAMs have robust cognition-enhancing effects in rodent models that are relevant for the cognitive deficits observed in schizophrenia patients.

**Discussion:**

These studies provide exciting new evidence that highly selective activators of two glutamate receptors identified in human genetic studies have potential utility in treatment of positive (mGLu1), negative (mGlu1), and cognitive (mGlu3) symptoms of schizophrenia patients. Furthermore, the novel mGlu1 and mGlu3 PAMs discovered in these studies provide excellent drug leads for further optimization and ultimate clinical testing.

